# Memantine Displays Antimicrobial Activity by Enhancing *Escherichia coli* Pathogen-Induced Formation of Neutrophil Extracellular Traps

**DOI:** 10.3389/fcimb.2020.00047

**Published:** 2020-02-13

**Authors:** Liang Peng, Li Li, Xiao-Long He, Jing-Yi Yu, Zhi-Jie Zeng, Wei-Jun Yang, Bao Zhang, Tie-Song Zhang, Hong Cao, Sheng-He Huang, Li-Qun Liu

**Affiliations:** ^1^Department of Clinical Laboratory, The Fifth Affiliated Hospital of Guangzhou Medical University, Guangzhou, China; ^2^Kunming Key Laboratory of Children Infection and Immunity, Yunnan Institute of Pediatrics, Kunming Children's Hospital, Kunming, China; ^3^Department of Pediatrics, Saban Research Institute, Childrens Hospital Los Angeles, University of Southern California, Los Angeles, CA, United States; ^4^Guangdong Provincial Key Laboratory of Tropical Diseases, Department of Microbiology, Southern Medical University, Guangzhou, China; ^5^Department of Pediatrics, The Second Xiangya Hospital, Central South University, Changsha, China

**Keywords:** bacterial infection, antibiotic resistance, memantine, neutrophil extracellular traps, α7 nAChR

## Abstract

Bacterial infection remains one of the leading causes of death worldwide due to the continuous rise of multiple antibiotic-resistant bacteria. Focusing solely on bacteria as the drug targets is a major limitation inherent in the conventional antibiotic therapy. Recently, host-directed therapies have become such an innovative approach to modulate the host defense system and the interplay of innate and adaptive immunity. Our previous studies showed that memantine (MEM), an α7 nAChR antagonist, could efficiently block multi-drug resistant *Escherichia coli*-caused bacteremia and meningitis in a mouse model. However, the underlying mechanisms that govern the antibacterial effects of MEM are still unknown. In this study, we demonstrated that MEM is able to significantly suppress *E. coli* infection by enhancing *E. coli*-induced formation and release of NETs *in vitro* and *in vivo*. MEM could promote the trapping and bactericidal activities of the polymorphonuclear neutrophils (PMNs) in a manner dependent on α7 nAChR, since knockdown of this receptor noticeably reduces the survival ability of bacteria in PMNs while MEM no longer affects the survival of bacteria in PMNs. Our results also showed that when the expression of S100A9, an antiseptic protein, is inhibited, pathogen survival rates in PMNs increase significantly. MEM reverses this effect in a concentration-dependent manner. MEM stimulates the production of MPO, S100A9, and DNA in PMNs and accelerates the release of depolymerized chromatin fibers into the extracellular space, suggesting the formation of NETs. Taken together, our data suggest that MEM effectively blocks bacterial infection through the promotion of the antibacterial function of NETs induced by *E. coli*.

## Introduction

The emergence of antibiotic resistance has become a severe public health problem. Bacteria resistant to multiple antibacterial agents such as carbapenem-resistant *enterobacteriaceae* (CRE), methicillin-resistant *Staphylococcus aureus* (MRSA), vancomycin-resistant *enterococci* (VRE), extensively drug-resistant *tuberculosis* (XDR-TB), and extensively drug-resistant *Acinetobacter baumannii* (XDRAB) are often referred to as “superbugs.” These bacteria infect at least 2 million people per year in the USA alone, with 23,000 dying as a direct result of these infections (Khan and Siddiqui, 2014). It suggests that there is an emergent need to develop new antibacterial drugs with novel strategies (Yu et al., 2015).

Host-directed therapies in adjunct to traditional antibiotic drugs become such innovative approaches to modulating the host defense system and the interplay of the innate and adaptive immunity (Munguia and Nizet, 2017). Development of a serious bacterial infection basically represents a failure of innate immune cells to execute their antimicrobial defense function (Munguia and Nizet, 2017). Pharmacologically targeting powerful immune cell killing and boosting the host defense system against pathogens could be an important way to treat infections, and would reduce frequencies in inducing drug resistance (Yu et al., 2015; Munguia and Nizet, 2017; Chiang et al., 2018).

Polymorphonuclear neutrophils (PMNs), the most abundant leukocytes in humans and other primates, play a central role in innate host defense against invading microorganisms (Hosoda et al., 2017). Activation of reactive oxygen species (ROS) is an important mechanism by which PMNs kill bacteria (Nguyen et al., 2017). Another important bacterial clearance pathway utilizes neutrophil extracellular traps (NETs), which are structures composed of granule proteins and nuclear constituents that are released by neutrophils. NETs have been shown to bind, disarm, and kill pathogens including both Gram-positive (*Staphylococcus aureus, Streptococcus pneumoniae*, and Group A streptococci), Gram-negative bacteria (*Salmonella enterica* erovar Typhimurium and *Shigella flexneri*), and certain fungi (*Candida albicans*). The granular components of NETs are peptides and enzymes (e.g., elastase and myeloperoxidase), and the nuclear constituents are chromatin DNA and histones (Brinkmann et al., 2004; Wartha et al., 2007b). Brinkmann et al. showed that neutrophils produce NETs when activated with interleukin-8 (IL-8), phorbolmyristate acetate (PMA), or lipopolysaccharide (Brinkmann et al., 2004).

Our previous studies on host-pathogen interplay show that α7 nAChR (α7R), an essential regulator of inflammation, is critical for the pathogenesis of *E. coli*-induced sepsis and meningitis (Chi et al., 2011, 2012). Using α7R KO *in vitro* and *in vivo* model systems, we have demonstrated that α7R plays a detrimental role in the genesis of bacteremia and the penetration of *E. coli* and neutrophils across the blood-brain barrier (BBB). These findings support the notion that α7R could serve as a unique drug target for broad-spectrum host-directed antimicrobial agents against bacterial infections, which lead to bacteremia and all too often sepsis. Using *in vitro* and *in vivo* models of the BBB and RNA-seq (Yu et al., 2015), our drug repositioning studies have shown that memantine (MEM), an FDA-approved drug for the treatment of Alzheimer's disease (AD), efficiently blocks pathogenicities induced by meningitic *Escherichia coli* E44 and IHE2015 (a multiple antibiotic-resistant strain) in a manner dependent on α7R. In addition, we found that MEM efficiently inhibits bacteremia caused by *E. coli* in an animal model (Wang et al., 2015; Yu et al., 2015).

Notably, NETs have been shown to be an important antibacterial mechanism, since NETs can capture microbial pathogens and exert bactericidal activity through the action of antimicrobial peptides, histone and other NET-associated components (Hosoda et al., 2017). Our laboratory has demonstrated that α7R is an essential regulator of the host inflammatory response against bacteria (Chi et al., 2011). α7R-mediated inflammatory effects could be blocked by its antagonist, MEM (Yu et al., 2015). Based on these findings, we hypothesized that MEM interacts first with the drug target α7R and then induce NET-mediated bacterial killing in PMNs. In this study we tried to confirm our hypothesis that the formation of NETs is associated with the ability of MEM to block infection by using *in vitro* and *in vivo* models.

## Materials and Methods

### Chemicals and Reagents

Memantine hydrochloride and the NETosis assay kit were purchased from Cayman Chemical (Ann Arbor, MI). The NET activator PMA, phagocytosis inhibitor Cytochalasin D, MPO antibody, and a S100A9 antibody were purchased from Abcam (Cambridge, MA). The neutrophil elastase (NE) ELISA Kit was purchased from R&D Systems (Minneapolis, MN). DNA fluorescent dye Picogreen was purchased from Thermo Fisher Scientific (Waltham, MA), *CHRNA7* (α7R encoding gene), and *S100A9* siRNA (small interfering RNA) kits were purchased from Santa Cruz Biotechnology (Santa Cruz, CA). The CHRNA7 antibody was purchased from GenScript (Piscataway, NJ). The S100A9 ELISA kit was purchased from CUSABIO (Wuhan, China).

### Bacterial Strains and Culture Conditions

*Escherichia coli* E44 is a rifampin-resistant strain derived from the RS218 strain (O18: K1: H7), which is a clinical isolate from the cerebrospinal fluid (CSF) of neonates with meningitis (Huang et al., 2001). RS218 and E44 have the same virulence phenotypes. E44-green fluorescent protein (GFP) is an E44 strain containing a GFP vector. Bacteria were cultured in L broth at 37°C overnight without agitation, unless otherwise specified.

### Detection of DNA and Neutrophil Elastase

The Ficoll-Paque method was used to isolate neutrophils from blood, and subsequently 1 × 10^5^ PMNs were cultured in each well of a 96-well-plate. The cells were then treated with 100 nm PMA, different concentrations of E44, or E44 along with MEM for 4 h. After the fluorescent DNA dye Picogreen was added and incubated with the samples for 10 min, the fluorescence value of every group was estimated with fluorescence microplate reader. Neutrophil elastase activity was measured using NETosis assay kit (Cayman Chemical) according to the manufacturer's instruction.

### Neutrophil Killing Assays

Neutrophil killing assays were performed as described by Katharina Beiter et al. (2006). A total of 1 × 10^6^ PMNs were seeded in 24 well-plates and cultured with RPMI medium (containing 10 mM HEPES and 2% heat-inactivated human serum) with or without Cytochalasin D, which is a phagocytosis inhibitor. The cells were treated with *E. coli* E44 (MOI = 50) and different concentrations of MEM (1, 5, 25 μM) for 2 h. The bacteria were then collected and enumerated after being cultured on LB agar plates containing rifampicin (30 μg/ml). Neutrophil killing was calculated on the basis of the number of colony-forming units (CFUs) recovered from wells with neutrophils and the CFUs recovered from wells without neutrophils. NET-mediated killing was calculated on the basis of the number of CFUs recovered from Cytochalasin-D-treated neutrophils and the CFUs recovered from Cytochalasin-D-treated wells without neutrophils. Intracellular killing was determined as the difference between total killing and extracellular killing.

Quantification of trapping was performed as described by Wartha et al. (2007a). Neutrophils were seeded and activated with 25 nM PMA for 10 min. After washing, cells were treated with 10 mg/ml Cytochalasin D to inhibit phagocytosis. An MOI of 50 was used for infection with *E. coli* E44, and different concentrations of MEM were incubated with the cells. After brief centrifugation and incubation for 5 min, the supernatant was removed and used for serial plating to quantify viable bacteria. The cells were subsequently treated for 15 min with RPMI ± DNase (20 U/ml) to dissolve NETs. Viable bacteria were quantified by the plating of serial dilutions. The percentage of CFUs trapped in NETs was calculated with the following formula: %trapped = [(CFU_Dnase_-CFU_RPMI_)/(CFU_Dnase_ + CFU_supernatant_)] × 100. CFU_DNase_ represents total numbers of untrapped and trapped bacteria since the DNase concentration of 20 U/ml did not affect bacterial viability. Under the condition without addition of DNase, the trapped bacteria were killed by NETs and the CFU_RPMI_ represent the viable untrapped bacteria. The relative trapping was calculated with the following formula: (%trapped in groups treated with MEM /% trapped in groups without MEM treatment) × 100.

To analyze DNA release from neutrophils induced by PMA, *E. coli*, or/and MEM, the DNA fluorescent dye Picogreen was added and incubated with the samples for 10 min, and the fluorescence value of every group was then estimated. Neutrophils without any treatment were used as the control group.

### *CHRNA7* and *S100A9* Knockdown Experiments

Knockdown experiments were performed using human CHRNA7 and S100A9 siRNA (small interfering RNA) kits from Santa Cruz Biotechnology as described previously (Ichikawa et al., 2011; Schaal et al., 2018). Opti-MEM (Gibco Life Technologies) and Lipofectamine 2000 transfection reagent (Invitrogen, USA) were used to transfect HL60 cells with siRNAs according to the supplier's recommendations. An unspecific scrambled siRNA was served as control. HL-60 cells were inoculated in 24-well plate and siRNA knockdown experiments were performed using a final siRNA concentration of 25 pmol with 2 μL transfection reagent per well. Media was replaced by complete medium containing 10% FBS 4–6 h after transfection. After replacing the medium and incubating for an additional 24 h, cells were used for western blot analysis, survival assays, and immunofluorescence assays.

### Human Neutrophil Isolation

Neutrophils were isolated from the peripheral blood of healthy donors as described by Halverson et al. (2015). The ethics committee of Southern Medical University (SMU) approved the study. Written informed consent was obtained from all participants. At first, whole blood was collected and mixed 5:1 in acid citrate dextrose. Red blood cells were then removed using dextran sedimentation and hypotonic lysis with KCl. The cell pellet was subjected to Ficol-Histopaque density centrifugation after the red blood cells had been lysed. The subsequent pellet was resuspended in 2 mL of HBSS (Hank's balanced salt solution, Invitrogen 14175-095). To determine the viable cell concentration, the collected cells were counted using a hemocytometer and Trypan blue staining.

### Immunofluorescence Microscopy

PMNs separated from blood were incubated with E44, MEM, and PMA. PMNs without any treatment were used as the control group. After blocking with 5% (w/v) BSA in PBS, the cells were stained with primary antibodies against S100A9 or MPO at 4°C overnight, then treated with a secondary antibody labeled with PE or FITC at room temperature for 1 h. The cells were immersed in mount medium containing DAPI, and examined under a fluorescence microscope at the Congressman Dixon Cellular Imaging Core Facility, Children's Hospital, Los Angeles. To ensure that the fluorescence strength of each treatment was comparable, all the images were acquired with the same parameters.

### Mice

C57BL/6J mice were obtained from the Animal Experimental Center of SMU. The animals were used at 6–8 weeks of age. All experiments were approved by the Animal Care and Ethics Committee of SMU. Mice (6 per group) were intraperitoneally injected with 2 × 10^7^ E44 or with 2 × 10^7^ E44 along with different concentrations of MEM (5–20 mg/kg of body weight). After infection with E44 for 24 h, mice were anesthetized and blood, liver, lung, and spleen tissue was collected. Blood samples were diluted and cultured on BHI agar plates containing rifampicin. Liver, lung, and spleen tissue was weighed, triturated, diluted, and cultured on BHI agar plates containing rifampicin.

### Statistical Analysis

The data were analyzed by ANOVA and covariates were followed by a multiple comparison test such as the Newmann-Keuls test to determine the statistical significance between the control and treatment groups. Graph Pad Prism v5.0 software was used. Comparisons with a *p* < 0.05 were considered to be statistically significant.

## Results

### MEM Promoted the E44-Induced Formation of NETs

**Figure 1:**
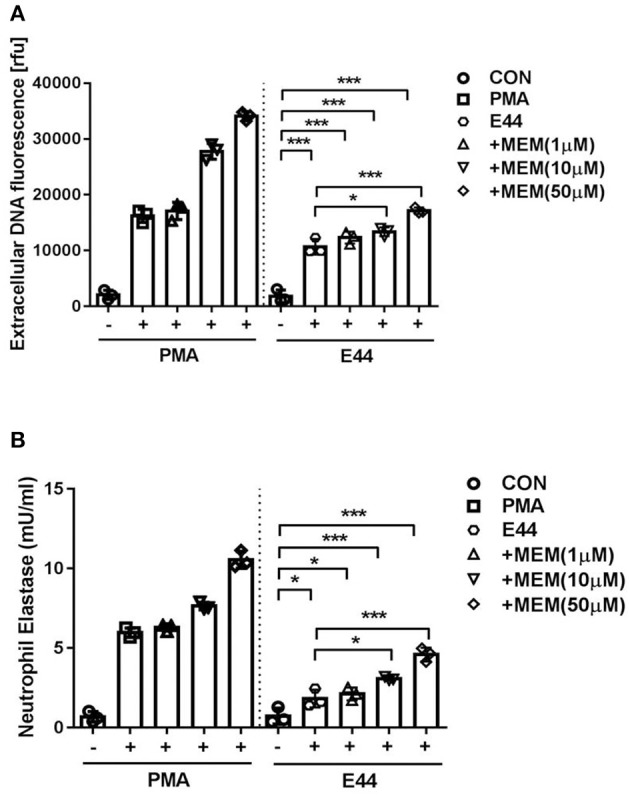
MEM enhanced E44-induced formation of NETs. **(A)** Detection of extracellular DNA using fluorescence microscope after the neutrophils infection with *E. coli* E44. A number of 1 × 10^5^ collected PMNs was cultured in 96 well-plates. The cells was treated with 100 nm PMA (positive control) or E44 for 4 h. Then the released DNA was detected with the DNA fluorescent dye of Picogreen; **(B)** The expression levels of neutrophil elastase (NE) in neutrophils treated with PMA or E44 along with MEM. Bar graphs show the means ± SD of three different experiments. Scatter plots in the bar graphs represent the three biological replicates. Significant differences with regard to the controls are marked by asterisks (*P < 0.05; ***P < 0.001).

DNA is a major component of NETs. Accordingly, we measured DNA release from neutrophils in order to assess NET formation. As shown in Figure 1A, MEM was able to enhance DNA release from PMNs infected with E44 in a concentration-dependent manner when compared to the control without treatment. Additionally, we measured the formation of neutrophil elastase (NE) using the NETosis assay kit. Similar to what was found when DNA release was analyzed, while E44 alone stimulated the production of NE, MEM was able to significantly promote E44-indcued NE release in a concentration-dependent manner (Figure 1B). These results indicate that MEM enhances E44-induced NET formation.

### MEM Promoted NET-Mediated Bacteria Trapping and Killing

**Figure 2:**
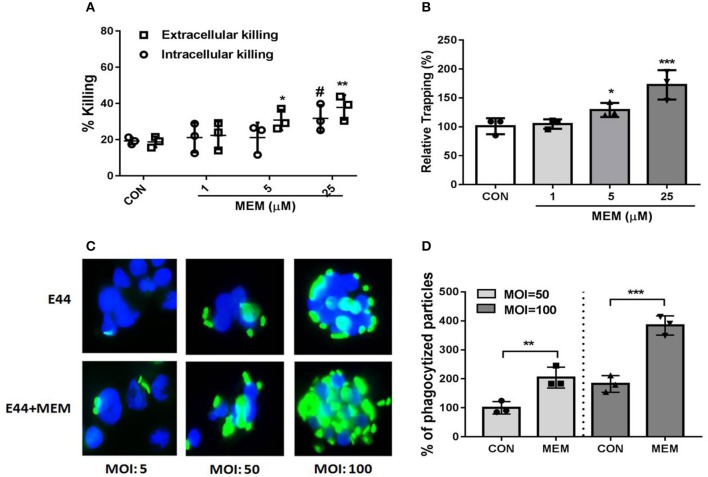
MEM enhanced bacterial killing by neutrophils. **(A)** Intracellular and extracellular killing of *E. coli* E44 after treatment with MEM. Neutrophil killing of bacteria includes two parts: intracellular killing and extracellular killing. A intracellular killing inhibitor of Cytochalasin D was used to distinguish intracellular killing of PMNs and extracellular killing microbes; **(B)** NETs trapping promoted by MEM. Neutrophils were stimulated for NET formation and infected with E44 at an MOI of 50. Different concentration of MEM was incubated with the neutrophils. Viable bacteria were quantified by plating of serial dilutions. The percentage of CFU trapped by NETs was calculated according to the formula described in section Materials and Methods; **(C)** MEM could enhance the intracellular killing ability of PMNs. Different MOI (5, 50, 100) was used for infection with E44-GFP, and a concentration of 50 μM MEM was added and incubated with PMNs for 1 h. PMNs without bacteria infection was used as a control group. Then the cells were collected by centrifuge and fixed on slide. DAPI was used for cell staining of nucleus (blue fluorescence). The intracellular killing of bacteria (green fluorescence) by neutrophils was counted, and 100 of neutrophils was assessed for each sample; **(D)** Phagocytized particles in PMNs treated with E44 or/and MEM. Phagocytized particles were counted under a fluorescence microscopy. CON represents cell treated with E44, and MEM group represents cell treated with E44 and memantine. Each bar represents the average of three different experiments ± SD (*n* = 3). Scatter plots in the bar graphs represent the three biological replicates. ^#^*P* < 0.05 (*P* value of the intracellular killing %), *P < 0.05, **P < 0.01, ***P < 0.001 (*P* value of the extracellular killing %).

Neutrophils kill bacteria either by phagocytosis (intracellular killing) or with NETs (extracellular killing). A phagocytosis inhibitor Cytochalasin D was used to distinguish intracellular killing and extracellular killing. A total of 1 × 10^6^ PMNs were cultured in 24-well plates with or without Cytochalasin D, and the cells were treated with E44 (MOI = 50) and different concentrations of MEM (0, 1, 5, and 25 μM) for 2 h. Subsequently, the bacteria were cultured on BHI agar plates, collected, and counted. As shown in Figure 2A, MEM significantly promoted the killing effect of PMNs on E44, including both intracellular killing and extracellular killing, in a concentration-dependent manner. Additionally, we found that when DNA was digested with DNase, MEM promoted NET trapping in a concentration-dependent manner (Figure 2B). As shown in Figures 2C,D, MEM was able to enhance the intracellular killing ability of PMNs. It is worth noting that when the MOI was <50, the PMNs did not show an obvious change in morphology. However, when the MOI was >50, each PMN was observed to contain at least 50 E44 bacteria, along with a significant nuclear morphology change. Collectively, these results suggest that MEM not only promotes the formation of NETs, but also enhances the NET-mediated bacterial trapping and killing of *E. coli* E44.

### MEM Enhanced the NET Function and Inhibits *E. coli* Dissemination *in vivo*

**Figure 3:**
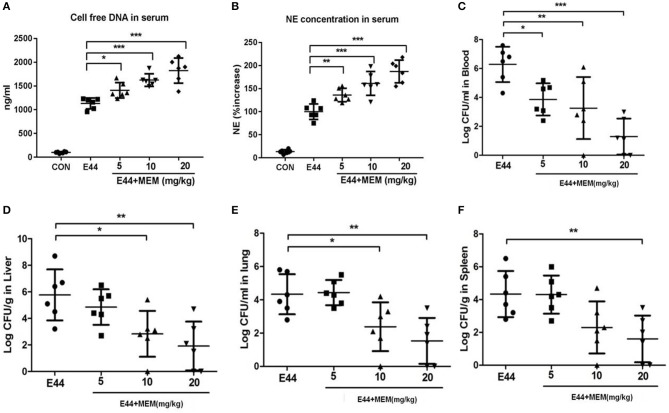
MEM promoted NETs formation and could block E44 dissemination in mouse model. Mice (6 per group) were intraperitoneally injected with 2 × 10^7^ E44 or with 2 × 10^7^ E44 along with different concentrations of MEM (5–20 mg/kg of body weight). After infection with E44 for 24 h, mice were anesthetized and blood, liver, lung, and spleen tissue was collected. The serum used to detect the cell free DNA using Picogreen and NE using a Mouse PMN Elastase ELISA kit. For NE detection, the serum was diluted 1:2. The blood was diluted from 1:10 to 1:10^8^ and cultured on BHI agar plates containing rifampicin. Liver, lung, and spleen tissue was weighed, triturated, diluted, and cultured on BHI agar plates containing rifampicin. **(A)** Cell free DNA in serum; **(B)** NE concentration in serum; **(C)** The bacterial levels in blood of mice; **(D)** The bacterial levels in liver tissue of mice; **(E)** The bacterial levels in lung tissue of mice; **(F)** The bacterial levels in spleen tissue of mice. The mice were treated as described in Materials and Methods, and then the blood, liver, lung, and spleen tissues were collected. Blood samples were diluted and cultured on the BHI agar plates. The liver, lung, and spleen tissues were weighted and triturated, then diluted and cultured on the BHI agar plates. All values represent the means of determinations. Bacteria levels in blood are expressed as log CFU/ml, and in tissues are expressed as log CFU/g. *P < 0.05, **P < 0.01, ***P < 0.001.

Mice were intraperitoneally injected with 2 × 10^7^ E44 or 2 × 10^7^ E44 along with different concentrations of MEM. Twenty four hours later, the mice were anesthetized and blood was collected. The serum was analyzed using Picogreen and the Mouse PMN elastase ELISA Kit (R&D Systems) to quantify the formation of NETs. As shown in Figures 3A,B, E44 significantly stimulated the release of DNA and elastase into blood. These results suggest that MEM promotes the formation of NETs *in vivo* after *E. coli* E44 infection.

To explore whether MEM blocks E44 dissemination, mice were intraperitoneally injected with 2 × 10^7^ E44 or 2 × 10^7^ E44 along with different concentrations of MEM. After infection with E44 for 24 h, the mice were anesthetized and blood, liver, lung, and spleen tissue was collected. Blood samples were diluted and cultured on BHI agar plates containing rifampicin. The liver, lung, and spleen tissue was weighed, triturated, diluted, and cultured on BHI agar plates containing rifampicin. As shown in Figures 3C–F, MEM effectively blocked the dissemination of E44 in infected mice.

### *CHRNA7* Knockdown Led to a Failure of MEM-Enhanced Intracellular Killing Ability of PMNs

**Figure 4:**
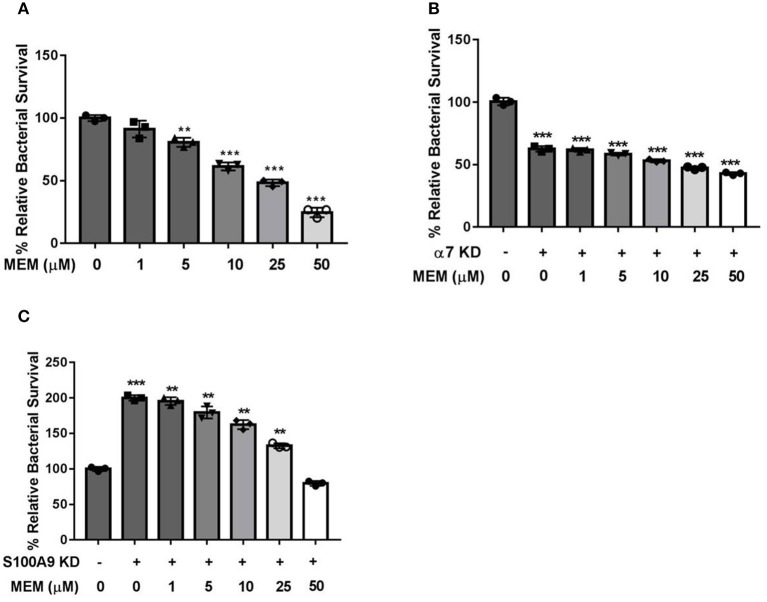
*CHRNA7* knockdown led to a failure of MEM-enhanced intracellular killing ability of PMNs. A total of 1 × 10^6^ of PMNs were cultured in 24-well plates and transfected with *CHRNA7* or *S100A9* siRNA. Cells were treated with *E. coli* E44 (MOI = 50) or/and different concentrations of MEM for 2 h. **(A)** Bacterial survival in PMNs with titration of memantine treatment; **(B)** Survival of E44 in PMNs with (+) or without (–) *CHRNA7* knockdown after treatment with different concentration of MEM; **(C)** Survival of E44 in PMNs with (+) or without (–) *S100A9* knockdown after treatment with different concentration of MEM. Relative survival was expressed as an n-fold increase or decrease relative to the basal level (Cells without siRNA and MEM treatment). Each bar represents the average of three different experiments ± SD. Scatter plots in the bar graphs represent the three biological replicates. **P < 0.01, ***P < 0.001 compared with the control.

S100A9 and α7R are both involved in the regulation of Ca^2+^-mediated signal transduction. S100A9 is also an antimicrobial protein which is released by PMNs during bacterial infection. HL60 cells were stimulated with 1.3% DMSO to generate PMNs. A total of 1 × 10^6^ PMNs of differentiated PMNs were cultured in 24-well plates and transfected with *CHRNA7* or *S100A9* siRNA using lipofectmine 2000. After the *CHRNA7* and *S100A9* genes were knocked down using siRNA, the intracellular killing ability of PMNs was tested (Figure 4A). demonstrated that MEM could enhance the intracellular killing ability of neutrophils in a manner of dose dependent. As shown in Figure 4B, the survival rate of E44 in the *CHRNA7* knockdown group was significantly lower than that of the normal group. The addition of MEM did not affect the intracellular killing ability of PMNs, even when the concentration of MEM reached 50 μM. As shown in Figure 4C, after knockdown of *S100A9*, the survival rate of E44 in PMNs increased when compared to the normal group.

### MEM Promoted the Expression of the Bactericidal Enzyme MPO and S100A9

**Figure 5:**
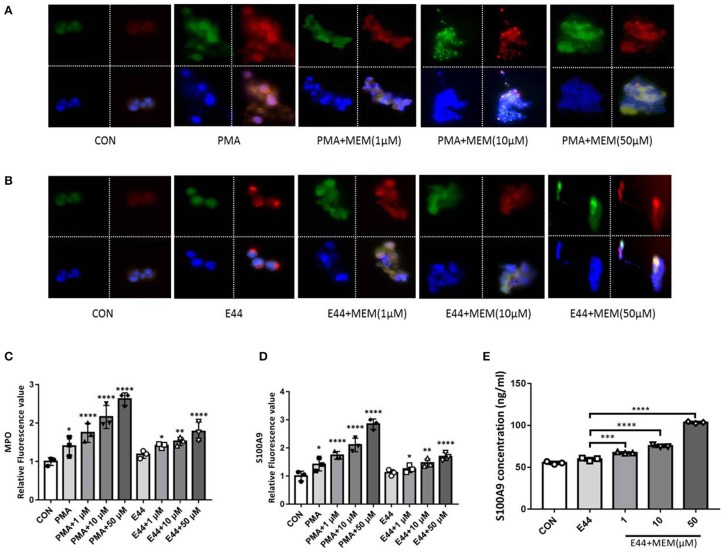
MEM promoted the expression of bactericidal enzyme MPO and antiseptic protein S100A9. The PMNs were treated with PMA, *E. coli* E44, different dose of MEM, and then incubated with fluorescent conjugated antibodies of MPO and S100A9, and the cell nucleus was stained with DAPI. **(A)** Expression of MPO and S100A9 in cells treated with PMA or PMA in combination with different concentration of MEM; **(B)** Expression of MPO and S100A9 in cells treated with E44 and different concentration of MEM; **(C)** Quantitative analysis of the relative fluorescence value of MPO induced by PMA, E44, and MEM; **(D)** Quantitative analysis of the relative fluorescence value of S100A9 induced by PMA, E44, and MEM; **(E)** Expression level of S100A9 in culture medium induced by E44 or/and MEM. A total of 1 × 10^6^ of PMNs were cultured in 24-well plates, and treated with *E. coli* E44 or/and different dose of MEM for 2 h. Then the samples were collected and centrifuged. The S100A9 in the supernatant was detected using the S100A9 ELISA kit according to the manufacturer's instructions. Each bar represents the average of three different experiments ± SD. Scatter plots in the bar graphs represent the three biological replicates. *P < 0.05, **P < 0.01, ***P < 0.001, ****P < 0.0001 compared with the control. Colors: MPO is shown in green; S100A9 is shown in red; Cell nucleus stained with DAPI is shown in blue.

PMNs were treated with PMA, *E. coli* E44, and different doses of MEM. Subsequently, these cells were analyzed using immunofluorescence. To detect the formation of NETs, all cell samples were incubated with fluorescent MPO (green) and S100A9 (red) antibodies, and nuclei were stained by DAPI. The fluorescence value was analyzed with Image J software, and the relative fluorescence value was calculated as the fluorescence values of treatment groups/the fluorescence value of control group. Under the fluorescence microscope, we observed that the nuclei of the control group cells maintained a segmented structure. However, the nuclear structures of cells in the groups treated with PMA, E44, and MEM were damaged significantly. These cells exhibited a mass production of MPO, S100A9, and DNA (Figures 5A–D). Additionally, depolymerized chromatin fibers were released into the extracellular space and formed a network structure, suggesting the formation of NETs. The expression level of S100A9 in cell culture medium induced by the MEM was also detected using ELISA method. The data as shown in Figure 5E demonstrated that MEM could significantly induce the expression of S100A9. Gene expression knockdown by transfection of siRNAs into PMNs weakened the promotion effects of E44 and MEM on the expression of MPO and S100A9 (Supplementary Data).

## Discussion

NETs have been considered to be a component of human innate immunity due to their ability to trap and kill pathogens. NETs are produced by activated neutrophils and consist of a DNA backbone with embedded antimicrobial peptides and enzymes. Granule proteins, histones, and chromatin together form an extracellular structure that traps and prevents the spread of bacteria (Brinkmann et al., 2004; Wartha et al., 2007b; Halverson et al., 2015). Proteases such as neutrophil elastase embedded in NETs degrade virulence factors (IpaB of *S. flexneri* and a toxin of *S. aureus*) (Weinrauch et al., 2002), bactericidal permeability-increasing protein (BPI), and histones. NETs also kill bacteria efficiently, and at least one of the NET components, histone, exerts antimicrobial activity at surprisingly low concentrations (Brinkmann et al., 2004).

In this study, we observed that pathogen-induced NET formation, including the release of elastase and DNA from neutrophils, was increased when the infected cells were incubated with MEM. In the mouse infection model, MEM could also induce the formation of NETs and inhibited the dissemination of *E. coli*. When compared to the control group, the distribution of bacteria in blood, liver, lung, and spleen tissues was significantly less in the MEM-treated group.

Previous reports suggest that α7R plays a detrimental role in the host defense against *E. coli* infection. It has been has confirmed that α7R is expressed on many types of cells, such as endothelial cells, fibroblasts, synoviocytes, polymorphonuclear neutrophils, dendritic cells, NK cells, B cells, and T cells (Tracey, 2009). Meningitic *E. coli* and nicotine can additively or synergistically induce the cellular release of Ca^2+^ which may expand bacterial cell signaling through the cholinergic α7R pathway (Chi et al., 2011). S100A9 is expressed abundantly in neutrophils and is able to regulate the ability of neutrophils to respond acutely to infection (Raquil et al., 2008; De Filippo et al., 2014; Yoshioka et al., 2016). The presence of S100A9 is critical in both murine and human NETs to inhibit bacterial growth (Achouiti et al., 2012). Results from the PMN intracellular killing assay indicate that the survival rate of E44 in the α7R knockdown group was significantly lower than that of the control group. Treatment with MEM did not affect the intracellular killing ability in this context. In contrast, after knockdown of S100A9, the bacterial survival rate in PMNs increased when compared to the control group. Furthermore, MEM inhibited bacterial survival in PMNs in which S100A9 was knocked down in a concentration-dependent manner. MEM could significantly stimulate the production of MPO, S100A9, and DNA in PMNs and accelerated the release of depolymerized chromatin fibers into the extracellular space, suggesting the formation of NETs.

Further investigation of factors that modulate the cytoplasmic EF-hand Ca^2+^-binding protein S100A9-mediated signaling pathway may reveal more insights into the mechanisms responsible for enhancement of neutrophil antimicrobial activity. S100A9 could be regulated by phosphorylation, but the importance of this phosphorylation on the NET activity of this protein has not yet been extensively studied (Schenten et al., 2018). Along these lines, it is interesting to determine if the enhancement of NET release and bactericidal activity is phosphorylation-dependent. Exploring the mechanism underlying phosphorylated S100A9-induced NET formation may facilitate discovery of signaling molecules specifically optimized to promote targeted enhancement of innate immune responses. Also, it is important to examine the impact of this phosphorylation on pro-inflammatory cytokine expression and secretion in neutrophils.

In conclusion, our data provide evidence that MEM is a host-directed antimicrobial agent that has the potential to be developed as a novel therapeutic for the treatment of bacterial infection. Furthermore, our data demonstrate a potential mechanism by which MEM exerts antimicrobial functions related to the promotion of pathogen-induced NET formation.

## Data Availability Statement

All datasets generated for this study are included in the article/Supplementary Material.

## Ethics Statement

The studies involving human participants were reviewed and approved by Ethics Committee of Southern Medical University. The patients/participants provided their written informed consent to participate in this study. The animal study was reviewed and approved by Animal Care and Ethics Committee of Southern Medical University.

## Author Contributions

S-HH, LP, L-QL, LL, J-YY, and X-LH conceived and designed the experiments. J-YY, LP, X-LH, LL, L-QL, Z-JZ, W-JY, and BZ performed the experiments. LP, X-LH, S-HH, J-YY, HC, and T-SZ analyzed the data. LP, S-HH, J-YY, X-LH, and HC contributed with reagents, materials, and analysis tools. LP, L-QL, S-HH, and J-YY wrote the paper.

### Conflict of Interest

The authors declare that the research was conducted in the absence of any commercial or financial relationships that could be construed as a potential conflict of interest.

## Abbreviations

MEM: Memantine
NETs: neutrophil extracellular traps
*E. coli*: *Escherichia coli*
α7R: α7 nAChR
MOI: multiplicity of infection
NE: neutrophil elastase
MPO: Myeloperoxidase
PMA: phorbolmyristate acetate.
