# Supplementary material for: Memantine Displays Antimicrobial Activity by Enhancing Escherichia coli Pathogen-Induced Formation of Neutrophil Extracellular Traps
Source: Front Cell Infect Microbiol. 2020 Feb 13;10:47. doi: 10.3389/fcimb.2020.00047 (PMC7031421; doi:10.3389/fcimb.2020.00047)
Supplement: Supplementary file 1 [file Data_Sheet_1.docx]

**Supplementary data**

Immunofluorescence analysis of *MPO* and *S100A9* knockdown

**A**

**
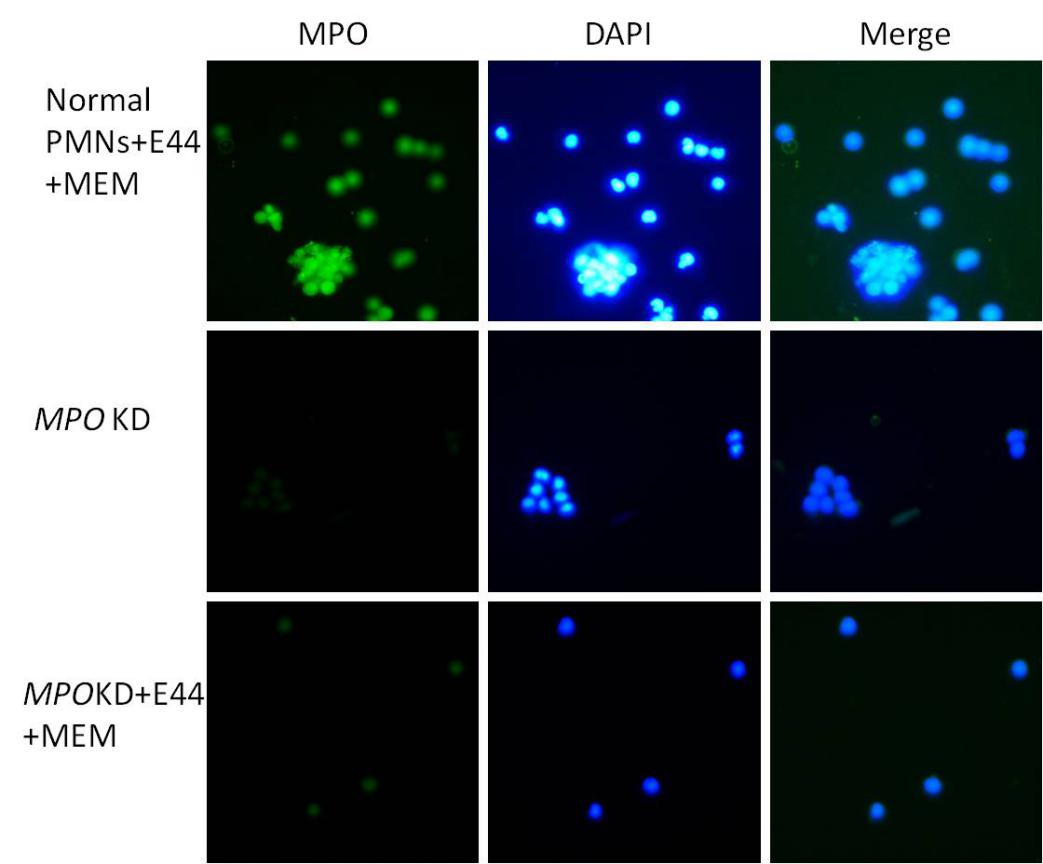
**

**B**

**
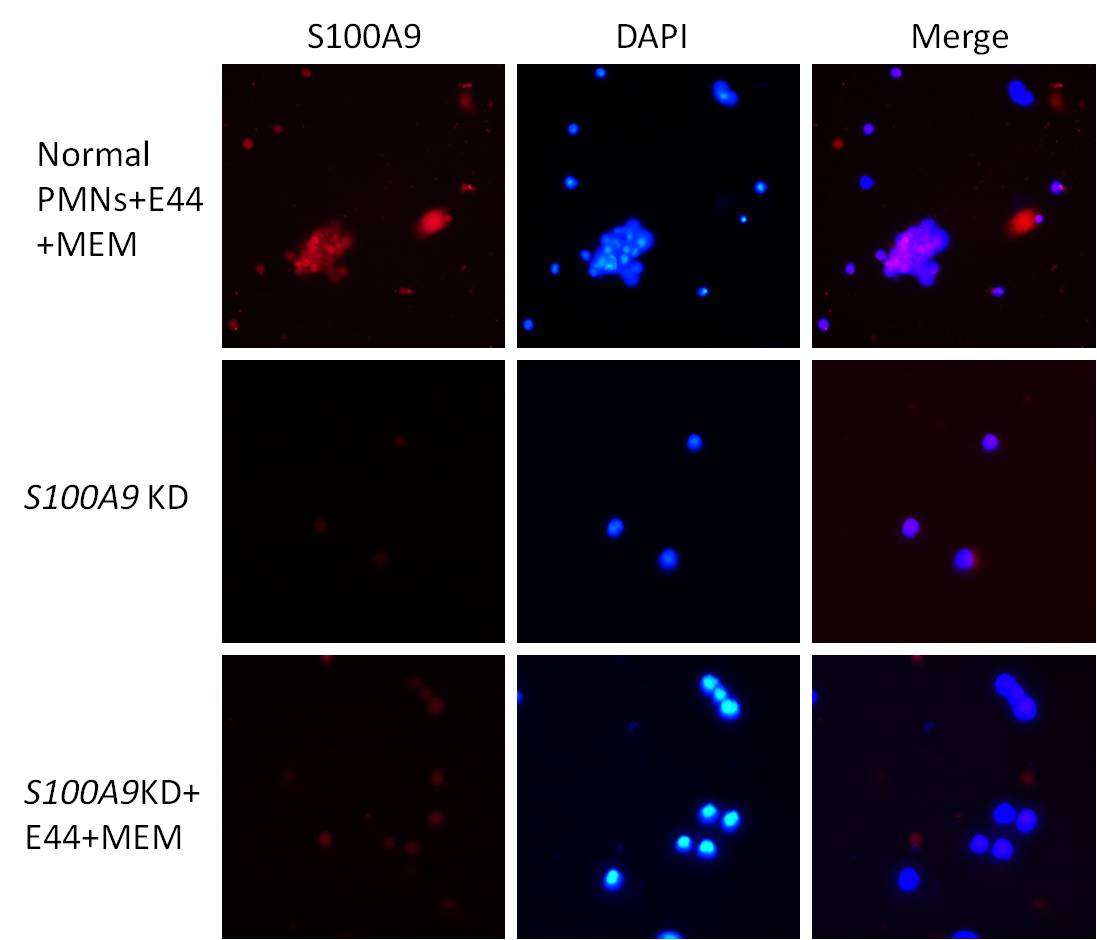
**

**C**


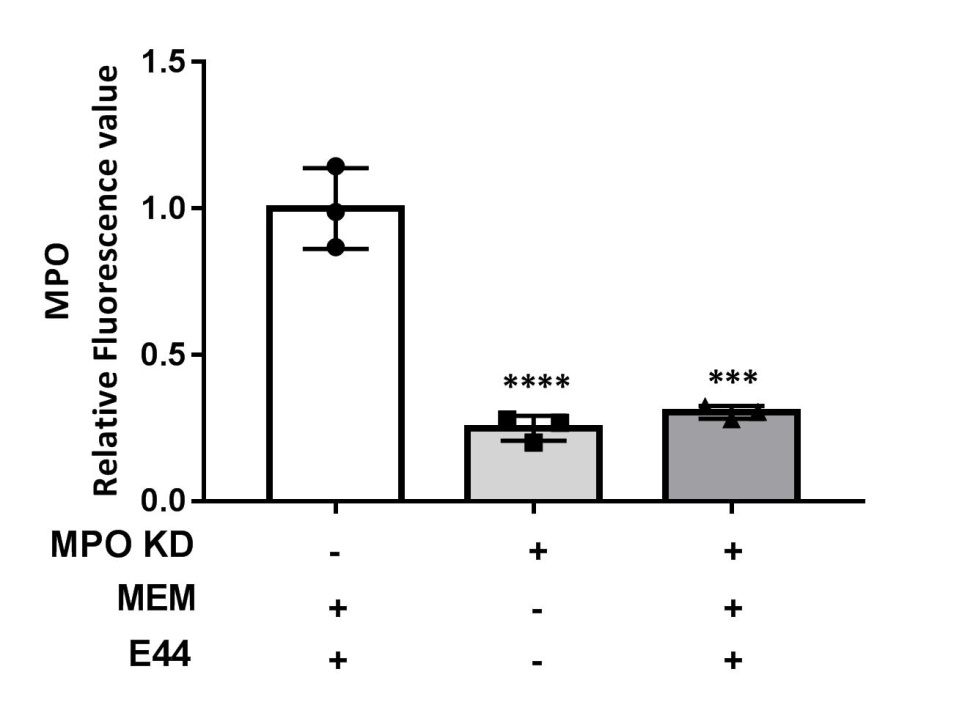


**D**


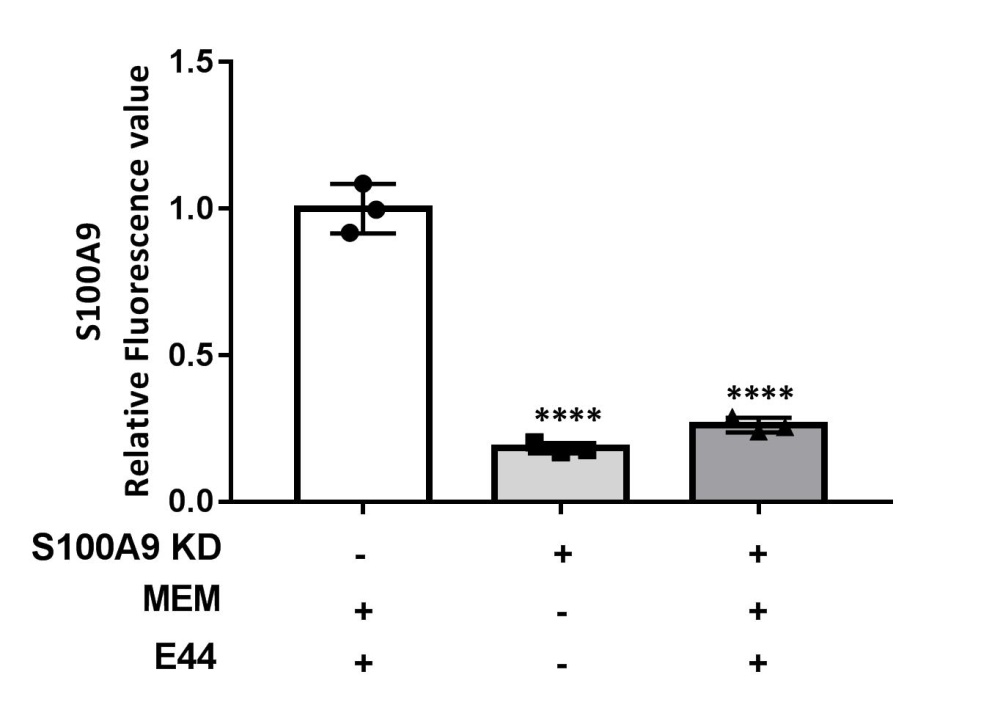


After being transfected with *MPO* or *S100A9* siRNA, the PMNs were treated with or without *E. coli* E44(MOI=50) and MEM (50μM). Cells were incubated with primary antibodies against S100A9 or MPO at 4°C overnight, then treated with a secondary antibody labeled with PE or FITC at room temperature for 1 h. The cell nucleus was stained with DAPI. (A) Expression of MPO and S100A9 in cells with or without siRNA transfection; (B) Quantitative analysis of the relative fluorescence value of MPO; (C) Quantitative analysis of the relative fluorescence value of S100A9. Each bar represents the average of three different experiments$\pm$SD. Scatter plots in the bar graphs represent the three biological replicates. ****P*<0.001 compared with the control; *****P*<0.0001 compared with the control.

Colors: MPO is shown in green; S100A9 is shown in red; Cell nucleus stained with DAPI is shown in blue.
